# Improvement of Vascular Function by Knockdown of Salusin-β in Hypertensive Rats via Nitric Oxide and Reactive Oxygen Species Signaling Pathway

**DOI:** 10.3389/fphys.2021.622954

**Published:** 2021-04-09

**Authors:** Yan Pan, Shuo Sun, Xingxing Wang, Aidong Chen, Xuejie Fei, Wei Wang, Ying Han

**Affiliations:** ^1^Key Laboratory of Targeted Intervention of Cardiovascular Disease, Collaborative Innovation Center of Translational Medicine for Cardiovascular Disease, Nanjing Medical University, Nanjing, China; ^2^Department of Physiology, Nanjing Medical University, Nanjing, China; ^3^Department of Emergency, Shanghai Putuo District People’s Hospital, Tongji University, Shanghai, China

**Keywords:** hypertension, salusin-β, vascular function, nitric oxide, reactive oxygen species

## Abstract

**Purpose:**

Salusin-β, a multifunctional vasoactive peptide, has a potentially important function in the pathological development of hypertension. However, the exact functional role of salusin-β and the underlying mechanism in this process are still not fully understood. The current study aimed to investigate the effects of silencing salusin-β on vascular function and vascular remodeling, as well as its signaling pathways in spontaneously hypertensive rats (SHR) and Wistar-Kyoto rats (WKY).

**Methods:**

Silencing salusin-β was performed by caudal vein injection of adenovirus expressing salusin-β short hairpin RNA (shRNA). Acetylcholine (ACh)-induced endothelium-dependent relaxation was used to evaluate vasodilator function, and high K^+^ solution-induced constriction was used to evaluate vasoconstriction function.

**Results:**

Salusin-β levels in plasma and its protein expression in mesenteric artery (MA), coronary artery (CA), and pulmonary artery (PA) of SHR were higher than those in WKY. The salusin-β level and expression were decreased effectively by salusin-β shRNA. Knockdown of salusin-β decreased arterial blood pressure (ABP) and high K^+^ solution-induced vascular constrictions, and improved the endothelium-dependent relaxation and vascular remodeling in SHR. The improved effect of silencing salusin-β on ACh-induced relaxation in SHR was almost blocked by the nitric oxide synthase (NOS) inhibitor L-NAME. Compared to WKY, the endothelial NOS (eNOS) activity and level, and nitric oxide (NO) level were decreased, while NAD(P)H oxidase activity and reactive oxygen species (ROS) levels in MA, CA, and PA of SHR were increased, which were all redressed by salusin-β knockdown.

**Conclusion:**

These results indicate that knockdown of salusin-β improves endothelium-dependent vascular relaxation and vascular remodeling and decreases ABP and vasoconstriction in SHR, which might be accomplished by increasing eNOS activation and NO release while inhibiting NAD(P)H oxidase derived-ROS generation. Scavenging salusin-β improves vascular function and then prevents the development and progression of vasculopathy of hypertension.

## Introduction

Primary hypertension accounts for the majority of hypertension cases, but its underlying pathological mechanism remains poorly understood. As a consequence, prevention and control of primary hypertension are still unsatisfactory (Chobanian et al., 2003; Gargiulo et al., 2015; Monticone et al., 2018). In many primary hypertensive patients and animal models, increased vascular constriction and attenuated vascular relaxation of small arteries due to endothelial dysfunction are two important hallmarks (Doyle, 1991; Higashi et al., 2002; Marik and Varon, 2007; Virdis et al., 2013). Acetylcholine (ACh) stimulates endothelial cells (ECs) to release nitric oxide (NO), prostacyclin (PGI_2_), and endothelium-derived hyperpolarizing factors (EDHF) to induce vascular smooth muscle cells (VSMCs) relaxation, so ACh is typically used to evaluate vascular endothelial function (Yuan et al., 1996; Wanstall et al., 2005; Raffetto et al., 2019). Among the three endothelium-derived relaxing factors, the bioavailability of NO plays the most important role in vascular relaxation. The reduction of vasorelaxation response to ACh was shown in 1987 in the small artery of spontaneously hypertensive rats (SHR; Carvalho et al., 1987). Recently, we also found that in SHR, ACh-induced vasodilatation of three important small arteries, the mesenteric artery (MA), coronary artery (CA), and pulmonary artery (PA), was significantly attenuated, which subsequently induced increases in peripheral resistance and blood pressure (Zhang et al., 2019a). With the pathological development of hypertension, the vascular function damage further contributes to heterogeneous vascular structure, and induces vascular remodeling (Laurent and Boutouyrie, 2015; Brown et al., 2018). Vascular remodeling includes an increase in vascular fibrosis, thickened and less elastic vascular walls, atherosclerosis, and stenosis of the arteries, which eventually causes complications in organs such as the heart, brain, and kidney, and it can even lead to death (Gueret et al., 2016; Ren X. et al., 2017; Zhang et al., 2019b). However, the mechanisms involved in increased vascular constriction, attenuated vascular relaxation, and vascular remodeling of small arteries in primary hypertension are still not very clear.

Salusin-β is an endogenous vasoactive peptide composed of 20 amino acid residues and belonging to the salusin family (Shichiri et al., 2003). It is closely related to cardiovascular disease (Sato et al., 2013; Kolakowska et al., 2015). Salusin-β is widely expressed in many tissues and cells, such as the central nervous system, immune system, endocrine system, peripheral vascular tissue, vascular ECs, and VSMCs (Suzuki et al., 2007, 2011; Sato et al., 2009). In patients with essential hypertension (Kolakowska et al., 2015) and some hypertensive animal models (Zhang et al., 2014), plasma salusin-β levels are significantly increased and are proportional to blood pressure. The previous studies have found that delivery of salusin-β to either the central paraventricular nucleus or rostral ventrolateral medulla increased renal sympathetic nerve activity, arterial blood pressure (ABP), and heart rate (HR) in rats (Chen et al., 2013; Sun et al., 2014; Li et al., 2015b; Xie et al., 2017). In addition, salusin-β causes endothelial dysfunction and injury in diabetes mellitus (Sun et al., 2017; Zhao et al., 2017) and provokes the NAD(P)H oxidase derived-reactive oxygen species (ROS) production in human umbilical vein ECs (Panieri and Santoro, 2015; Sag et al., 2017; Yin et al., 2017; Esfahani et al., 2018). However, the effects of silencing salusin-β expression on vascular function in primary hypertensive rats remain unexplored.

The present study is therefore designed to evaluate the effects of silencing the salusin-β gene on high blood pressure, increased vascular constriction, attenuated endothelium dependent relaxation, and vascular remodeling of three important arteries, MA, CA, and PA in SHR, and to explore the downstream molecular mechanisms of salusin-β activity in SHR.

## Materials and Methods

All animals were housed in a temperature- and humidity-controlled animal room on a 12 h light-dark cycle with free access to standard chow and tap water. The procedures were approved by Nanjing Medical University Experimental Animal Care and complied with the Guide for the Care and Use of Laboratory Animals published by the US National Institutes of Health (NIH publication, 8th edition, 2011). Wistar-Kyoto rats (WKY) were used as the control for SHR, and 13–14-week-old male adult SHR and WKY rats (Vital River Laboratory Animal Technology Co., Ltd., Beijing, China) were used in this experiment.

### Salusin-β Knockdown

The adenoviral vectors encoding salusin-β small hairpin RNA (shRNA) and control shRNA were constructed by Genomeditech Co. (Shanghai, China) according to our previous reports (Sun et al., 2016b; Zhao et al., 2017). Caudal vein injection of phosphate-buffered saline (PBS), adenovirus expressing control shRNA or salusin-β shRNA (2 × 10^11^ plaque forming units/mL) was performed in three groups of WKY and three groups of SHR. After 2 weeks, specific knockdown of salusin-β was verified by measurement of salusin-β levels in plasma by ELISA and protein expression in arteries of rats by using both western blot and immunohistochemistry.

### Arterial Blood Pressure Measurement in a Conscious State

The ABP of the tail artery of conscious rats was measured with a non-invasive computerized tail-cuff system (Kent Scientific Corporation, CT, United States). The rats were trained by measuring ABP daily for at least 10 days before the formal testing to minimize stress-induced ABP fluctuations. Before the measurements, the rats were warmed for 10–20 min at 28°C to allow for detection of tail arterial pulsations and to achieve the steady pulse. Then an annular tube connected with the transducer was put at the root of the rat tail. After the tail arterial pulsation waveforms were observed, the rubber annular tube on the tail was inflated and then deflated to get the systolic blood pressure (SBP). The diastolic blood pressure (DBP) and HR in the conscious state were calculated by software. The blood pressure was obtained by averaging 10 measurements.

### ABP Recording Under Anesthesia

The right carotid artery of rat anesthetized by intraperitoneal injection with urethane (800 mg/kg) was cannulated and connected to a pressure transducer (MLT0380, ADInstruments, Australia) linked to a Powerlab data acquisition system (8SP, ADInstruments, Australia) to continuously record ABP. Then, the SBP, DBP, mean arterial pressure, and HR under anesthesia were calculated.

### Arteries Sample Preparation

For the purpose of detecting salusin-β protein expression by western blot or immunohistochemistry, eNOS activity, eNOS level, NO level, NAD(P)H oxidase activity, and superoxide anion levels of arteries, third-order MA, CA or PA samples were isolated from rats, flash-frozen in liquid nitrogen, and stored at -70°C. Then, the artery tissues were homogenized in RIPA lysis buffer supplemented with protease inhibitor and centrifuged. The total proteins in the homogenate supernatant were extracted and measured using a protein assay kit (BCA, Pierce, United States).

### Measurement of Salusin-β Protein Expression in Arteries

Western blot method was used to measure the salusin-β protein expression in arteries as described in previous reports (Li et al., 2015a, 2019; Zhao et al., 2017). Briefly, proteins in the artery tissue homogenate supernatant were separated by a 10% SDS–PAGE gel first and transferred to a nitrocellulose membrane. The membrane was then probed with anti-salusin-β IgG (1:1000, Cloud-Clone Corp, United States) followed by incubation with horseradish peroxidase-conjugated goat anti-rabbit IgG (1:5000; Immunology Consultants Lab, Portland, OR, United States). The bands were visualized with an enhanced chemiluminescence ECL system (Pierce Chemical, Rockford, IL, United States). The β-actin antibody (1:5000; Abways Technology Inc, Shanghai, China) protein was used as the loading control. The total amounts of salusin-β protein were normalized to the β-actin protein.

### Salusin-β Immunohistochemistry

Salusin-β immunohistochemistry of the arteries was performed with an immunohistochemistry kit (Abcam, MA, United States). Briefly, the arteries were processed into coronal sections (5 μm) and incubated with anti-salusin-β IgG (1:1000, Cloud-Clone Corp, United States) at 4°C overnight. After washing, sections were incubated with biotinylated goat anti-rabbit IgG for 1 h and then stained with DAB according to the manufacturer’s instructions. Sections were covered with mounting medium, and salusin-β immunoreactivity was observed under a light microscope (DP70, Olympus, Tokyo, Japan).

### Measurement of Salusin-β Level in Plasma, eNOS Activity, eNOS Level, and NO Level

The salusin-β level in plasma and eNOS level of arteries were detected by enzyme immunoassay kits (Cloud-clone Corp., Wuhan, China and Yi Fei Xue Biotechnology, Nanjing, China) following the manufacturer’s instructions. The activity of eNOS in arteries was assessed by the conversion of L-arginine to NO using the Nitric Oxide Synthase Assay Kit (Beyotime Biotech Inc., Nanjing, China). NO production in arteries was evaluated with the Nitrate/Nitrite Colorimetric Assay Kit (Cayman Chemical Co., Ann Arbor, MI, United States).

### Measurement of NAD(P)H Oxidase Activities and Superoxide Anion Levels

NAD(P)H oxidase activity and superoxide anion levels of arteries were measured by enhanced lucigenin-derived chemiluminescence, as described in our previous research (Ren X. S. et al., 2017). Briefly, for superoxide anion levels measurement, the light emissions produced by the reactions between lucigenin (5 μmol/L) and the superoxide anions in tissue homogenate supernatant were measured by a luminometer (20/20n, Turner, CA, United States) once every minute for 10 min. For NAD(P)H oxidase activity measurement, NAD(P)H (100 μmol/L) was added to the medium as a substrate to react with NAD(P)H oxidase to generate superoxide anions before the reactions between lucigenin and superoxide anions were detected by the luminometer. The values represent NAD(P)H oxidase activity and superoxide anion levels are expressed as the mean light unit (MLU) per minute per milligram of protein.

### Isometric Tension Measurements in Arteries

Arterial contraction and relaxation function were measured by isometric tension experiments as previously reported (Han et al., 2015; Zhang et al., 2019a). Briefly, the third-order MA, CA, and PA were isolated from rats and cut into 1- to 1.2-mm segments in Krebs-Henseleit solution [the components of it were as previously described (Zhang et al., 2019a)]. Arterial rings (one arterial ring/artery/rat was used) were mounted to the jaw in a four-chambered myograph (620M, DMT, Denmark) and set at a resting tension of 0.1 g as previously reported (Estrada et al., 2012; Han et al., 2015). After equilibration, high K^+^ solution [the concentration of K^+^ inside of it was 0.15 mol/L as previously described (Zhang et al., 2019a)] was added into chamber to induce arterial ring contraction to evaluate arterial contracting function, and then was washed. Afterward, the prostaglandin F2α (PGF 2α; 1–5 μmol/L; Estrada et al., 2012) was used to induce arterial ring contraction, followed by six doses of ACh (10^–9^∼10^–4^ mol/L) administration in a dose-dependent manner to induce vascular relaxation as previously reported (Freiman et al., 1986; White et al., 2000). The contractile responses induced by PGF 2α are similar between WKY and SHR and have no significant differences among MA, CA, and PA. The degree of relaxation is shown as a percentage of PGF 2α-induced contraction. The pretreatment of arteries with chemicals, such as L-NAME (10^–2^ mol/L), INDO (10 μmol/L), TEA (10^–2^ mol/L), and VAS2870 (10 μmol/L), was performed 20 min before PGF 2α.

### Evaluation of Vascular Remodeling

Third-order coronary arteries, pulmonary arteries, or mesenteric arteries were isolated from rats, embedded in paraffin, cut into 5-μm thick cross-sections, and stained with hematoxylin-eosin (HE) staining. Structural changes of these arteries were observed with a light microscope. The media thickness, lumen diameter, and media thickness/lumen diameter were used as indexes of vascular remodeling (Bagnost et al., 2010).

### Chemicals

Prostaglandin F2α, ACh, NAD(P)H, N’-nitro-L-arginine-methyl ester hydrochloride (L-NAME), dimethyl sulfoxide (DMSO), indomethacin (INDO), and tetraethylammonium (TEA) were purchased from Sigma Chemical Co. (St. Louis, MO, United States). VAS2870 (NADPH oxidase inhibitor) was purchased from Abcam (Cambridge, MO, United States). The chemicals were dissolved in normal saline except for VAS2870 and INDO, which were dissolved in DMSO.

### Statistical Analysis

Data are expressed as the mean ± S.E. One-way or two-way ANOVA was used, followed with Bonferrioni *post hoc* test to compare with the multiple groups. *P* < 0.05 was considered statistically significant. The pD2 and Emax data of the ACh-induced relaxation curves were calculated by GraphPad Prism software.

## Results

### Effects of Salusin-β Knockdown on Salusin-β Levels in Plasma and Protein Expression in Arteries

**FIGURE 1:**
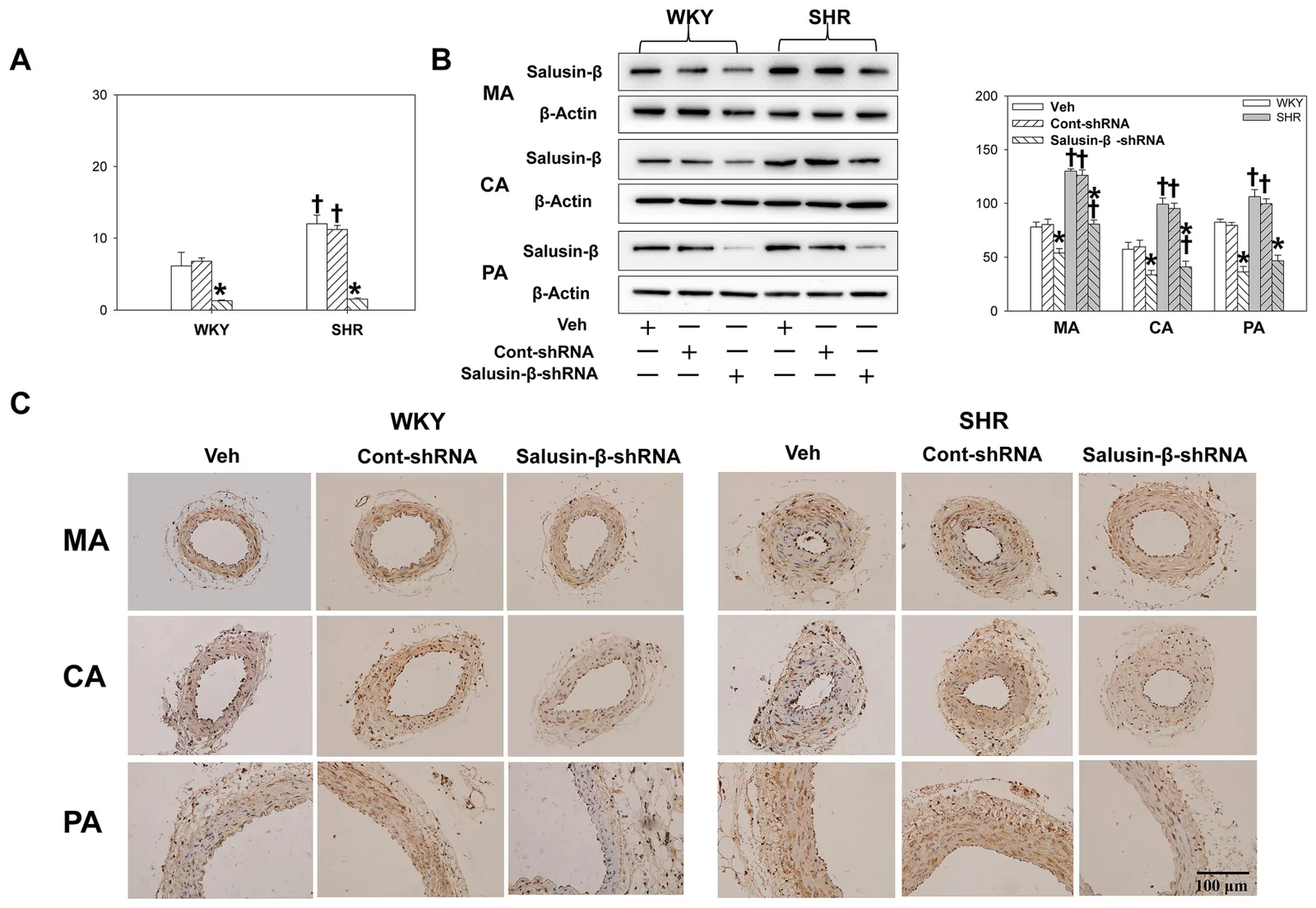
The salusin-β level in plasma **(A)**, and salusin-β protein expression of MA, CA, and PA in WKY and SHR detected by western blot **(B)** and detected by immunohistochemistry **(C)** after salusin-β knockdown. Two-way ANOVA was used for data analysis, followed by Bonferrioni *post hoc* test to compare with the multiple groups. Values are mean ± SE. ^∗^*P* < 0.05 compared with Veh or Cont-shRNA, ^†^*P* < 0.05 compared with WKY. *n* = 6 for each group.

Compared with WKY, the salusin-β levels in plasma (Figure 1A) and the salusin-β protein expressions in MA, CA, and PA of SHR detected by either western blot (Figure 1B) or immunohistochemistry (Figure 1C) were increased. Both salusin-β levels in plasma and protein expression in MA, CA, and PA of WKY and SHR were decreased significantly after intravenous injection of adenoviral vectors encoding salusin-β shRNA to knockdown salusin-β (Figure 1).

### Effects of Salusin-β Knockdown on Blood Pressure

The SBP and DBP of SHR measured either in conscious state or under anesthesia were much higher than those of WKY. After salusin-β knockdown, both SBP and DBP of SHR were decreased; furthermore, the decrease in DBP was more significant. The HR of SHR measured in conscious state was slightly higher than that of WKY, which was eliminated by salusin-β knockdown. There was no significant difference in body weight between WKY and SHR (Table 1).

**TABLE 1:** Effects of salusin-β knockdown on body weight, SBP, DBP, and HR in WKY and SHR in conscious state or under anesthesia.

	**WKY**			**SHR**		
	**Veh**	**Cont-**	**Salusin-β-shRNA**	**Veh**	**Cont-**	**Salusin-β-shRNA**
Body weight, *g*	325.1 ± 5.3	320.6 ± 4.9	319.9 ± 5.1	323.7 ± 4.2	321.4 ± 4.8	327.6 ± 5.3
SBP in conscious state, mm Hg	118.4 ± 4.0	119.5 ± 3.1	117.9 ± 4.4	204.4 ± 3.9^†^	205.7 ± 4.1^†^	185.2 ± 4.4*^†^
DBP in conscious state, mm Hg	80.3 ± 2.8	82.2 ± 3.7	78.9 ± 4.2	162.0 ± 4.6^†^	165.0 ± 4.8^†^	137.1 ± 5.1*^†^
HR in conscious state, beats/min	373.7 ± 13.3	365.4 ± 11.1	376.4 ± 12.9	439.9 ± 8.2^†^	440.6 ± 11.1^†^	393.4 ± 14.2*
SBP under anesthesia, mm Hg	106.2 ± 3.8	108.4 ± 4.1	105.7 ± 3.9	186.5 ± 6.8^†^	184.7 ± 5.9^†^	166.5 ± 4.2*^†^
DBP under anesthesia, mm Hg	78.6 ± 3.3	79.7 ± 4.2	77.9 ± 3.1	149.5 ± 7.7^†^	145.2 ± 6.4^†^	109.9 ± 5.1*^†^
HR under anesthesia, beats/min	376.8 ± 14.5	380.4 ± 13.8	372.1 ± 15.6	392.8 ± 16.0	385.3 ± 14.9	389.3 ± 15.5

SBP, systolic blood pressure; DBP, diastolic arterial pressure; and HR, heart rate. SBP, DBP, and HR of tail artery were measured in conscious state by use of a non-invasive computerized tail-cuff system. SBP, DBP, and HR of right carotid artery under anesthesia were measured with a pressure transducer. Values are expressed as mean ± SE. *P < 0.05 vs. Veh or Cont-shRNA. ^†^*P* < 0.05 vs. WKY. *n* = 6 for each group.

### Effects of Salusin-β Knockdown on High K + Induced Vascular Contraction

The high K^+^ solution induced vasoconstrictions in MA, CA, and PA were enhanced significantly in SHR compared to WKY. After salusin-β knockdown, the vasoconstriction of SHR induced by high K^+^ was decreased significantly, while it was almost unchanged in WKY. There was no significant difference between the PBS (Veh) and Control shRNA (Cont-shRNA) groups (Table 2).

**TABLE 2:** Effects of salusin-β knockdown on the high K^+^ induced contraction (mg/mm) in MA, CA, and PA in WKY and SHR.

	**WKY**			**SHR**		
	**Veh**	**Cont-**	**Salusin-β-shRNA**	**Veh**	**Cont-**	**Salusin-β-shRNA**
MA	341.5 ± 27.4	316.3 ± 29.7	350.6 ± 28.5	806.7 ± 29.9^†^	771.7 ± 22.4^†^	352.1 ± 27.9*
CA	162.6 ± 17.7	167.1 ± 18.2	156.0 ± 12.0	301.4 ± 21.8^†^	306.9 ± 21.1^†^	175.9 ± 22.1*
PA	198.8 ± 22.6	206.0 ± 26.9	218.7 ± 16.6	355.8 ± 25.7^†^	343.5 ± 30.2^†^	222.3 ± 24.2*

Values are expressed as mean ± SE. *P < 0.05 vs. Veh or Cont-shRNA. ^†^*P* < 0.05 vs. WKY. *n* = 6 for each group.

### Effects of Salusin-β Knockdown on Endothelium-Dependent Vascular Relaxation

**FIGURE 2:**
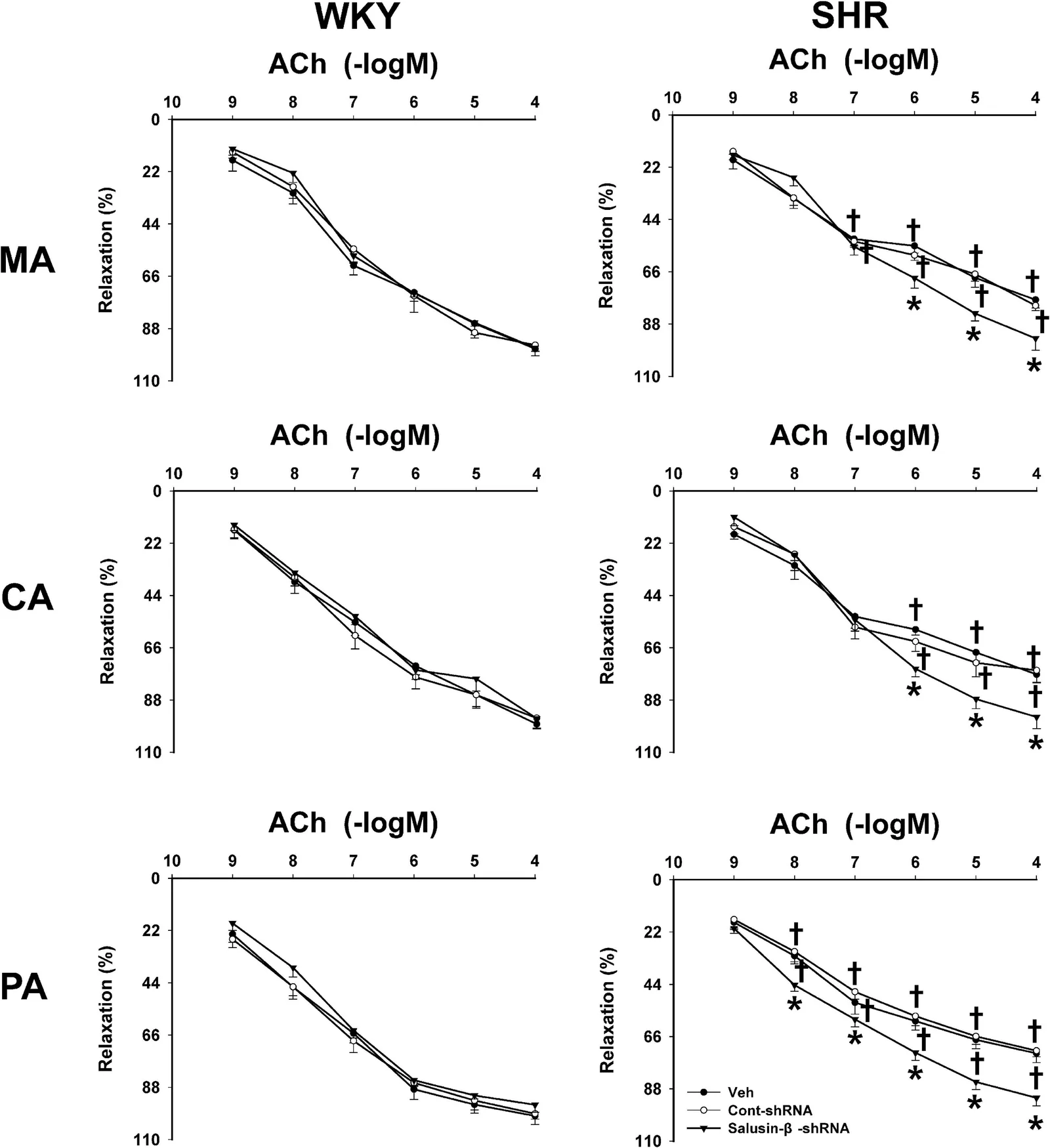
The effects of salusin-β knockdown on ACh-induced dose-dependent relaxation in MA, CA, and PA in WKY and SHR. One-way ANOVA was used for data analysis in MA, CA, and PA of WKY rats, and two-way ANOVA was used for data analysis in SHR rats, followed by Bonferrioni *post hoc* test to compare with the multiple groups. Values are mean ± SE. ^∗^*P* < 0.05 compared with Veh or Cont-shRNA, ^†^*P* < 0.05 compared with WKY. *n* = 6 for each group.

Compared to WKY, ACh-induced dose-dependent relaxations in MA, CA, and PA of SHR were attenuated significantly, while they were improved to a nearly normal physiological state by salusin-β knockdown in SHR. Silencing salusin-β had no significant effect on endothelium-dependent vascular relaxation in WKY (Figure 2). The pD2 and Emax data of ACh-induced dose-dependent vascular relaxation in MA, CA and PA in WKY and SHR treated with Veh, Control-shRNA or salusin-β-shRNA were displayed in Table 3, which also supported the above findings.

**TABLE 3:** The Emax (%) and pD2 (-logEC50) data of ACh-induced dose-dependent vascular relaxation for each group of treatments on MA, CA, and PA in WKY and SHR.

		**MA**			
		**WKY**		**SHR**	
		**pD2**	**Emax**	**pD2**	**Emax**
Veh		7.16 ± 0.19	96.5 ± 1.05	7.11 ± 0.34	77.8 ± 3.43^†^
Cont-shRNA		7.13 ± 0.24	95.0 ± 2.50	7.22 ± 0.21	80.2 ± 2.19^†^
Salusin-β-shRNA		7.18 ± 0.30	96.2 ± 3.23	7.14 ± 0.66	94.0 ± 4.99*^‡^
Cont-shRNA	Saline	7.11 ± 0.25	97.5 ± 2.05	7.15 ± 0.42	78.2 ± 2.19^†^
	TEA	6.91 ± 0.17	87.4 ± 3.11*	6.91 ± 0.31	81.3 ± 3.03
	L-NAME	Not converged	6.8 ± 5.33*	Not converged	16.6 ± 4.12*
	0.1%DMSO	7.27 ± 0.32	98.5 ± 3.05	7.23 ± 0.27	77.8 ± 2.43^†^
	INDO	7.28 ± 0.16	85.4 ± 3.50*	6.92 ± 0.30	76.8 ± 3.77
	VAS2870	7.28 ± 0.19	100.9 ± 2.01	7.16 ± 0.31	87.4 ± 3.04*^†^
Salusin-β-shRNA	Saline	7.08 ± 0.21	94.2 ± 2.26	7.24 ± 0.43	95.6 ± 3.12^‡^
	TEA	6.83 ± 0.23	83.4 ± 2.52*	7.39 ± 0.19	92.6 ± 1.40^‡^
	L-NAME	Not converged	3.4 ± 6.52*	Not converged	12.6 ± 7.12*
	0.1%DMSO	7.14 ± 0.34	96.2 ± 1.26	7.18 ± 0.14	93.0 ± 1.16^‡^
	INDO	7.22 ± 0.17	90.5 ± 1.78	6.94 ± 0.25	91.5 ± 1.58^‡^
	VAS2870	7.18 ± 0.29	102.9 ± 4.87	7.78 ± 0.23	96.5 ± 1.20^‡^

		**CA**			
		**WKY**		**SHR**	
		**pD2**	**Emax**	**pD2**	**Emax**

Veh		7.18 ± 0.26	98.1 ± 1.84	7.22 ± 0.27	77.2 ± 3.61^†^
Cont-shRNA		7.23 ± 0.25	95.6 ± 4.50	7.20 ± 0.15	75.5 ± 4.98^†^
Salusin-β-shRNA		7.09 ± 0.17	95.8 ± 4.01	7.05 ± 0.39	95.1 ± 4.90^*‡^
Cont-shRNA	Saline	7.21 ± 0.25	98.6 ± 2.50	7.16 ± 0.22	75.2 ± 2.61^†^
	TEA	7.24 ± 0.28	91.7 ± 3.24	6.90 ± 0.47	63.2 ± 2.51^*†^
	L-NAME	Not converged	9.7 ± 5.24^∗^	Not converged	4.6 ± 6.12^∗^
	0.1%DMSO	7.27 ± 0.32	99.6 ± 1.50	7.07 ± 0.39	73.5 ± 2.61^†^
	INDO	7.31 ± 0.33	85.7 ± 1.69^∗^	6.86 ± 0.16	72.4 ± 3.87^†^
	VAS2870	7.15 ± 0.17	94.7 ± 1.52	7.37 ± 0.19	84.4 ± 2.35^*†^
Salusin-β-shRNA	Saline	7.17 ± 0.33	96.8 ± 3.01	7.13 ± 0.37	95.1 ± 2.90^‡^
	TEA	7.03 ± 0.35	88.7 ± 1.52	7.10 ± 0.19	85.2 ± 1.31^*‡^
	L-NAME	Not converged	7.4 ± 4.52^∗^	Not converged	2.6 ± 4.12^∗^
	0.1%DMSO	7.19 ± 0.26	98.8 ± 3.01	7.37 ± 0.47	97.9 ± 1.19^‡^
	INDO	7.15 ± 0.36	86.5 ± 3.18^∗^	7.25 ± 0.20	86.8 ± 3.46^*‡^
	VAS2870	7.35 ± 0.23	99.3 ± 3.87	7.24 ± 0.33	94.0 ± 1.97^‡^

		**PA**			
		**WKY**		**SHR**	
		**pD2**	**Emax**	**pD2**	**Emax**

Veh		7.23 ± 0.24	100.0 ± 3.63	7.25 ± 0.28	73.1 ± 3.85^†^
Cont-shRNA		7.20 ± 0.27	99.1 ± 2.23	7.31 ± 0.30	72.0 ± 2.55^†^
Salusin-β-shRNA		7.21 ± 0.10	95.4 ± 3.12	7.29 ± 0.24	91.8 ± 3.36^*‡^
Cont-shRNA	Saline	7.18 ± 0.30	97.1 ± 1.23	7.14 ± 0.20	73.0 ± 3.55^†^
	TEA	7.10 ± 0.16	84.3 ± 2.89^∗^	6.35 ± 0.26^∗^	72.6 ± 2.34^†^
	L-NAME	Not converged	14.3 ± 5.89^∗^	Not converged	6.6 ± 5.12^∗^
	0.1%DMSO	7.23 ± 0.28	96.1 ± 3.23	7.18 ± 0.27	74.0 ± 3.55^†^
	INDO	7.27 ± 0.12	85.8 ± 2.72^∗^	7.28 ± 0.12	73.9 ± 4.34
	VAS2870	7.19 ± 0.06	95.3 ± 0.86	7.26 ± 0.12	84.4 ± 3.35^*†^
Salusin-β-shRNA	Saline	7.11 ± 0.22	96.4 ± 2.12	7.29 ± 0.30	91.8 ± 2.36^‡^
	TEA	6.99 ± 0.35	87.3 ± 3.89	7.41 ± 0.23	86.3 ± 2.34^‡^
	L-NAME	Not converged	11.4 ± 3.52^∗^	Not converged	10.6 ± 5.37^∗^
	0.1%DMSO	7.21 ± 0.29	97.4 ± 3.12	7.13 ± 0.23	91.9 ± 2.07^‡^
	INDO	7.05 ± 0.21	84.8 ± 3.78^∗^	7.12 ± 0.29	83.0 ± 2.32^∗^
	VAS2870	7.31 ± 0.28	97.8 ± 2.86	7.51 ± 0.26	93.2 ± 2.91

Values are mean ± SE. *P < 0.05 vs. Veh or Saline or 0.1%DMSO. ^†^*P* < 0.05 vs. WKY. ^‡^*P* < 0.05 vs. Cont-shRNA. *n* = 6 for each group.

### Effects of Salusin-β Knockdown on Vascular Remodeling

**FIGURE 3:**
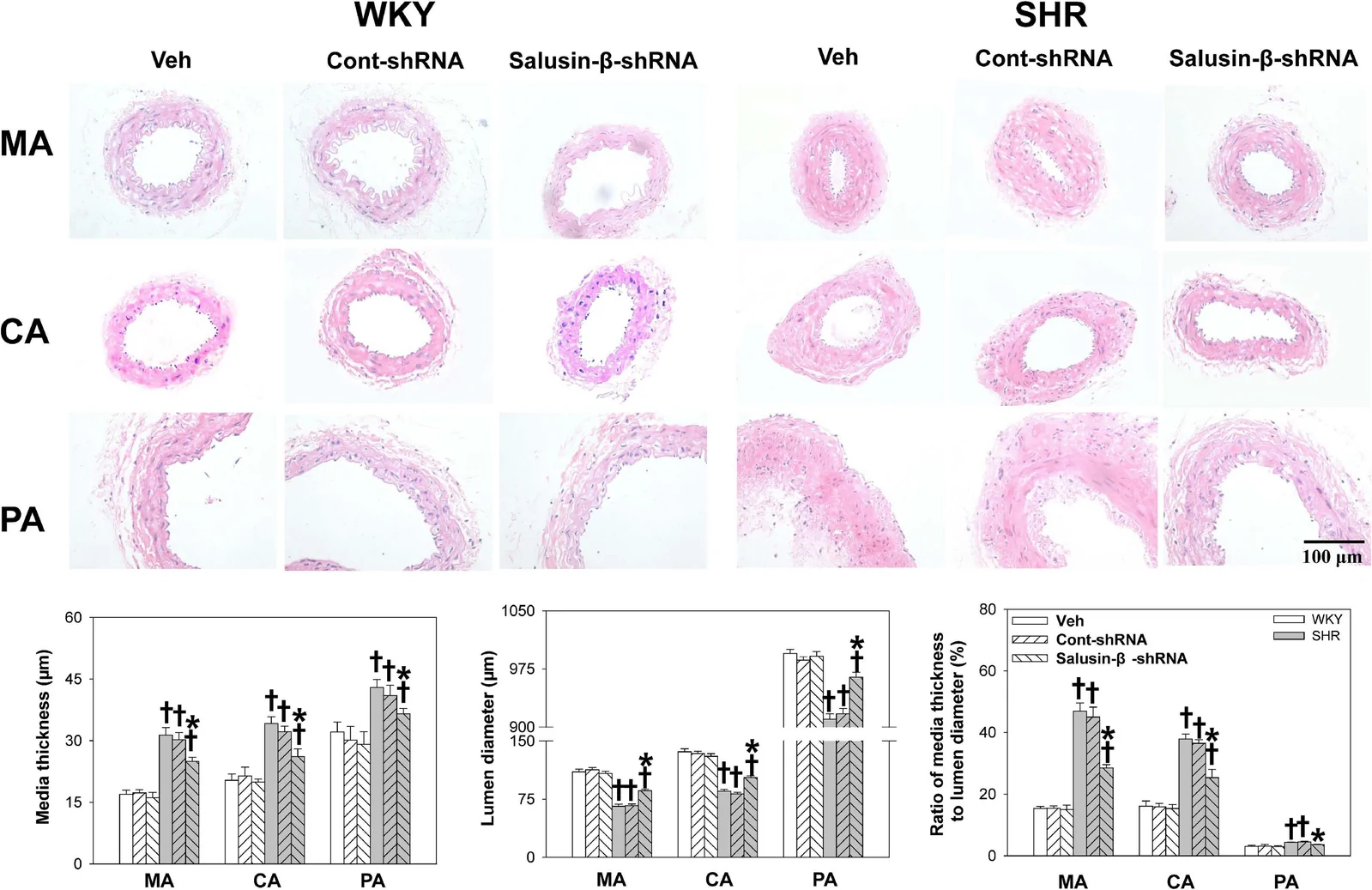
The media thickness, lumen diameter, and media thickness/lumen diameter of MA, CA, and PA in WKY and SHR after salusin-β knockdown. Two-way ANOVA was used for data analysis, followed by Bonferrioni *post hoc* test to compare with the multiple groups. Values are mean ± SE. ^∗^*P* < 0.05 compared with Veh or Cont-shRNA, ^†^*P* < 0.05 compared with WKY. *n* = 6 for each group.

Compared with WKY, the lumen diameter of MA, CA, and PA in SHR decreased, while media thickness and media thickness/lumen diameter increased significantly, which indicated the occurrence of vascular remodeling in SHR. Salusin-β knockdown increased the lumen diameter and decreased the media thickness and media thickness/lumen diameter of MA, CA, and PA in SHR, suggesting that vascular remodeling in SHR was restored by salusin-β knockdown (Figure 3).

### Effects of L-NAME, INDO, and TEA on Vascular Relaxation Response to Salusin-β Knockdown

**FIGURE 4:**
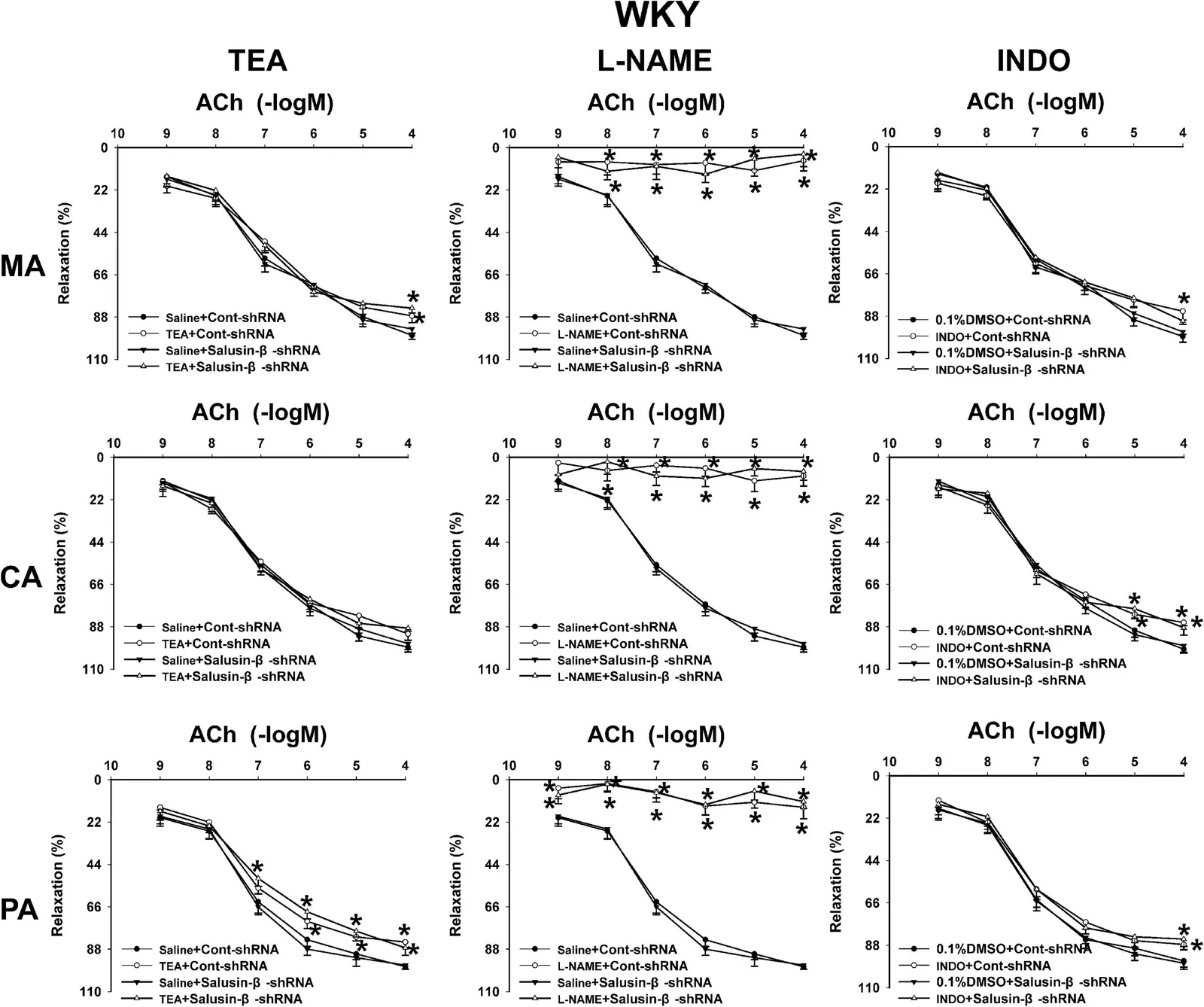
The effects of L-NAME, INDO, and TEA on the ACh-induced vascular relaxation in MA, CA, and PA in WKY treated with Cont-shRNA or salusin-β-shRNA. One-way ANOVA was used for data analysis, followed by Bonferrioni *post hoc* test to compare with the multiple groups. Values are mean ± SE. ^∗^*P* < 0.05 compared with Saline or 0.1% DMSO. *n* = 6 for each group.

**FIGURE 5:**
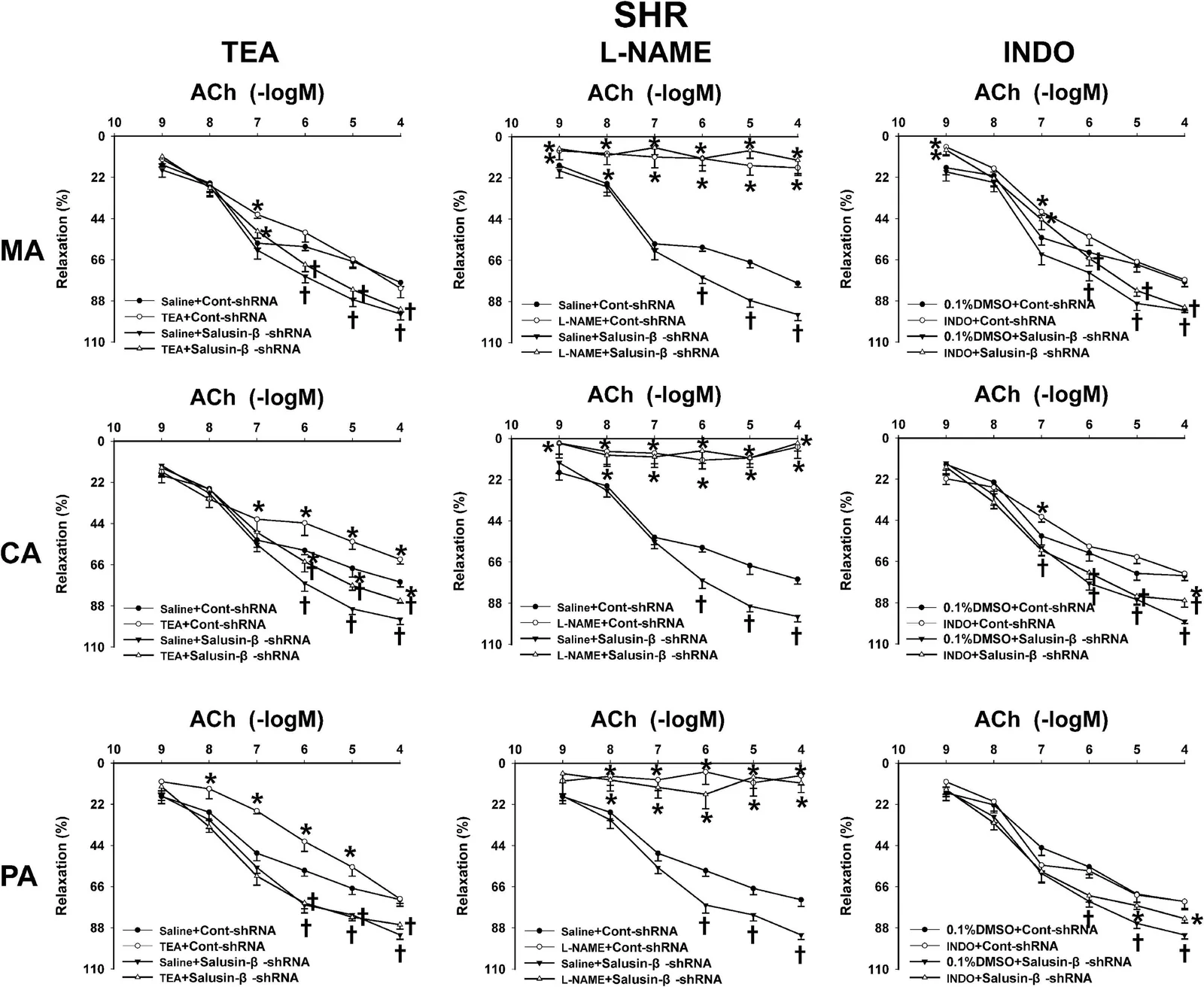
The effects of L-NAME, INDO, and TEA on the ACh-induced vascular relaxation in SHR treated with Cont-shRNA or salusin-β-shRNA. One-way ANOVA was used for data analysis, followed by Bonferrioni *post hoc* test to compare with the multiple groups. Values are mean ± SE. ^∗^*P* < 0.05 compared with Saline or 0.1% DMSO, ^†^*P* < 0.05 compared with Cont-shRNA. *n* = 6 for each group.

The nitric oxide synthase (NOS) inhibitor L-NAME almost abolished the ACh-induced dose-dependent relaxations in MA, CA, and PA in both WKY (Figure 4) and SHR (Figure 5) with or without salusin-β knockdown. While the pretreatment of TEA, the K^+^ channels inhibitor, or INDO, the non-specific inhibitor of PGI_2_ synthesis, on arteries attenuated slightly the ACh-induced dose-dependent relaxations in both WKY and SHR with or without salusin-β knockdown. There was no significant difference between the effects of DMSO and saline (Figures 4, 5). The pD2 and Emax data of ACh-induced dose-dependent vascular relaxation in MA, CA, and PA for each treatment in WKY and SHR are displayed in Table 3.

### Effects of VAS2870 on Vascular Relaxation Response to Salusin-β Knockdown

**FIGURE 6:**
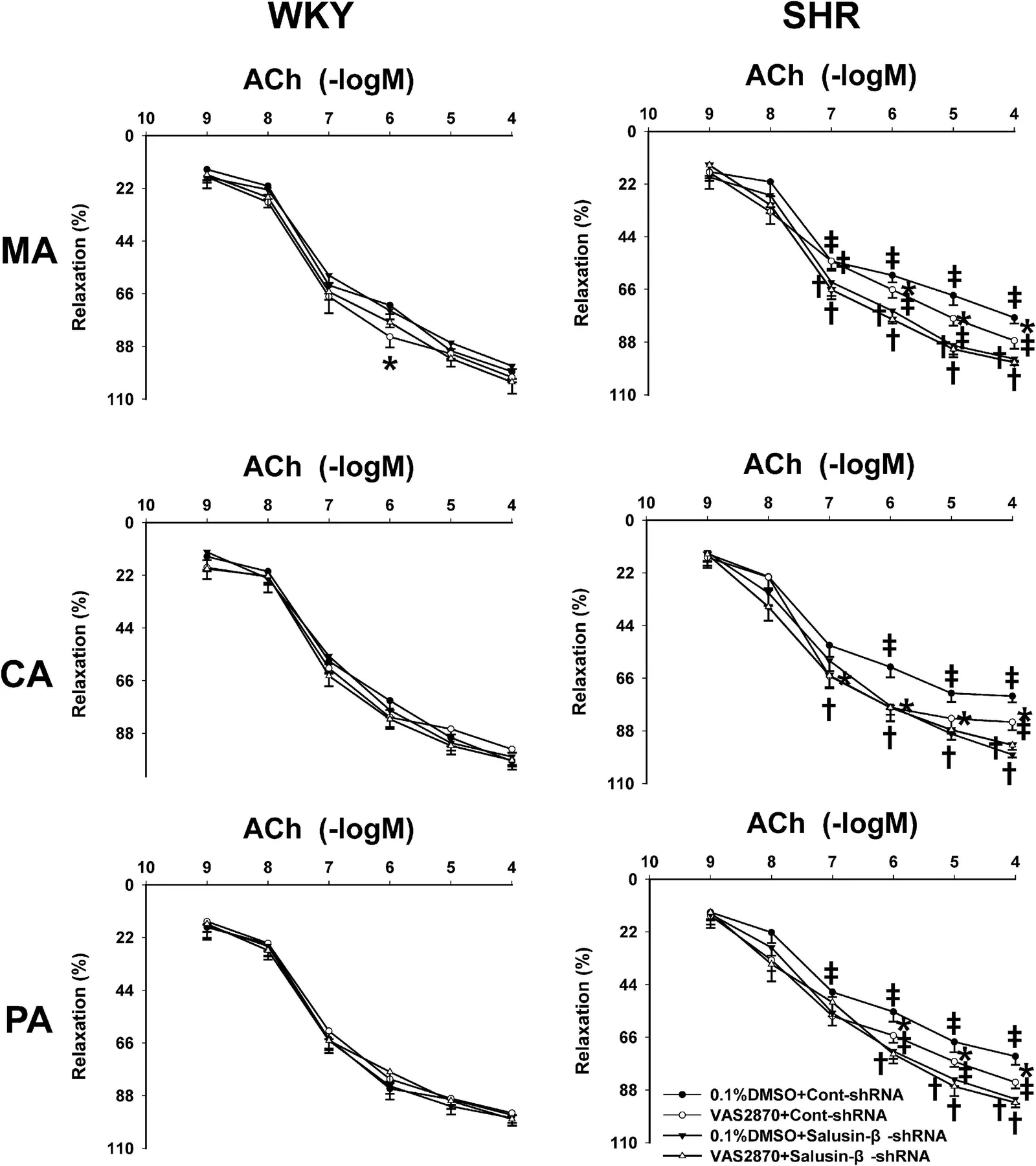
The effects of VAS2870 on ACh-induced vascular relaxation response to salusin-β knockdown in MA, CA, and PA in WKY and SHR. Two-way ANOVA was used for data analysis, followed by Bonferrioni *post hoc* test to compare with the multiple groups. Values are mean ± SE. ^∗^*P* < 0.05 compared with 0.1% DMSO, ^†^*P* < 0.05 compared with Cont-shRNA. ^‡^*P* < 0.05 compared with WKY. *n* = 6 for each group.

VAS2870 is an NAD(P)H oxidase inhibitor that is effective in suppressing the activation of NAD(P)H oxidase and subsequent production of intracellular ROS (Altenhofer et al., 2015). VAS2870 improved ACh-induced endothelium-dependent relaxations in MA, CA, and PA in SHR with Cont-shRNA, which is similar to the effects of salusin-β knockdown on vascular relaxation. However, VAS2870 had no significant effect on endothelium-dependent vascular relaxation in WKY and SHR treated with salusin-β-shRNA (Figure 6). The pD2 and Emax data of ACh-induced dose-dependent vascular relaxation in MA, CA, and PA in WKY and SHR are displayed in Table 3.

### Effects of Salusin-β Knockdown on eNOS Level, eNOS Activity, and NO Level

**FIGURE 7:**
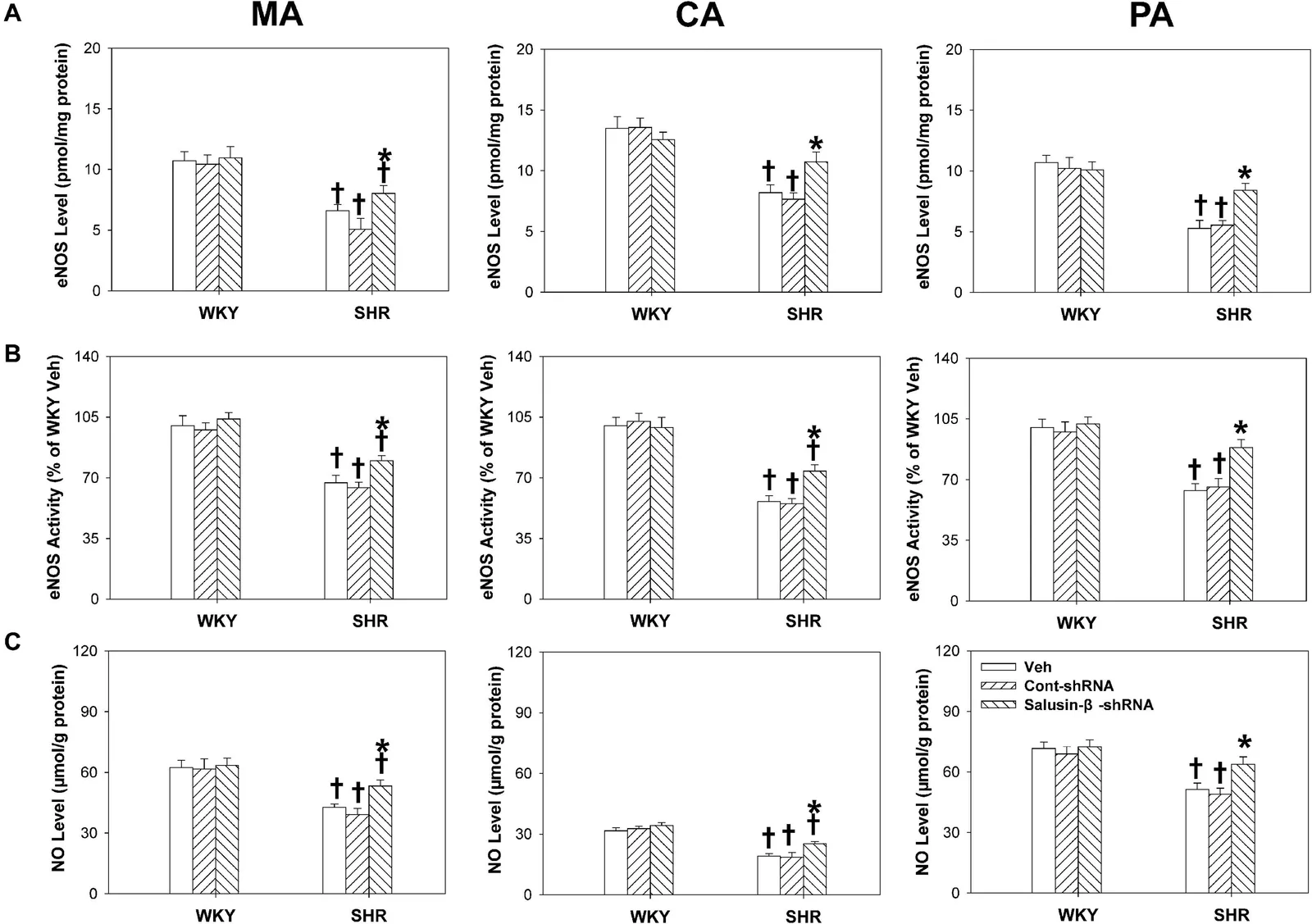
The effects of salusin-β knockdown on eNOS level **(A)**, eNOS activity **(B)** and NO level **(C)** of MA, CA, and PA in WKY and SHR. Two-way ANOVA was used for data analysis, followed by Bonferrioni *post hoc* test to compare with the multiple groups. Values are mean ± SE. ^∗^*P* < 0.05 compared with Veh or Cont-shRNA, ^†^*P* < 0.05 compared with WKY. *n* = 6 for each group.

The eNOS level (Figure 7A), eNOS activity (Figure 7B), and NO level (Figure 7C) of MA, CA, and PA of SHR were much lower than those of WKY. After salusin-β knockdown, the eNOS level, eNOS activity, and NO level of arteries of SHR increased. However, silencing salusin-β had no significant effect on eNOS activity and NO level in WKY (Figure 7).

### Effects of Salusin-β Knockdown on NAD(P)H Oxidase Activity and Superoxide Anion Levels in Arteries

**FIGURE 8:**
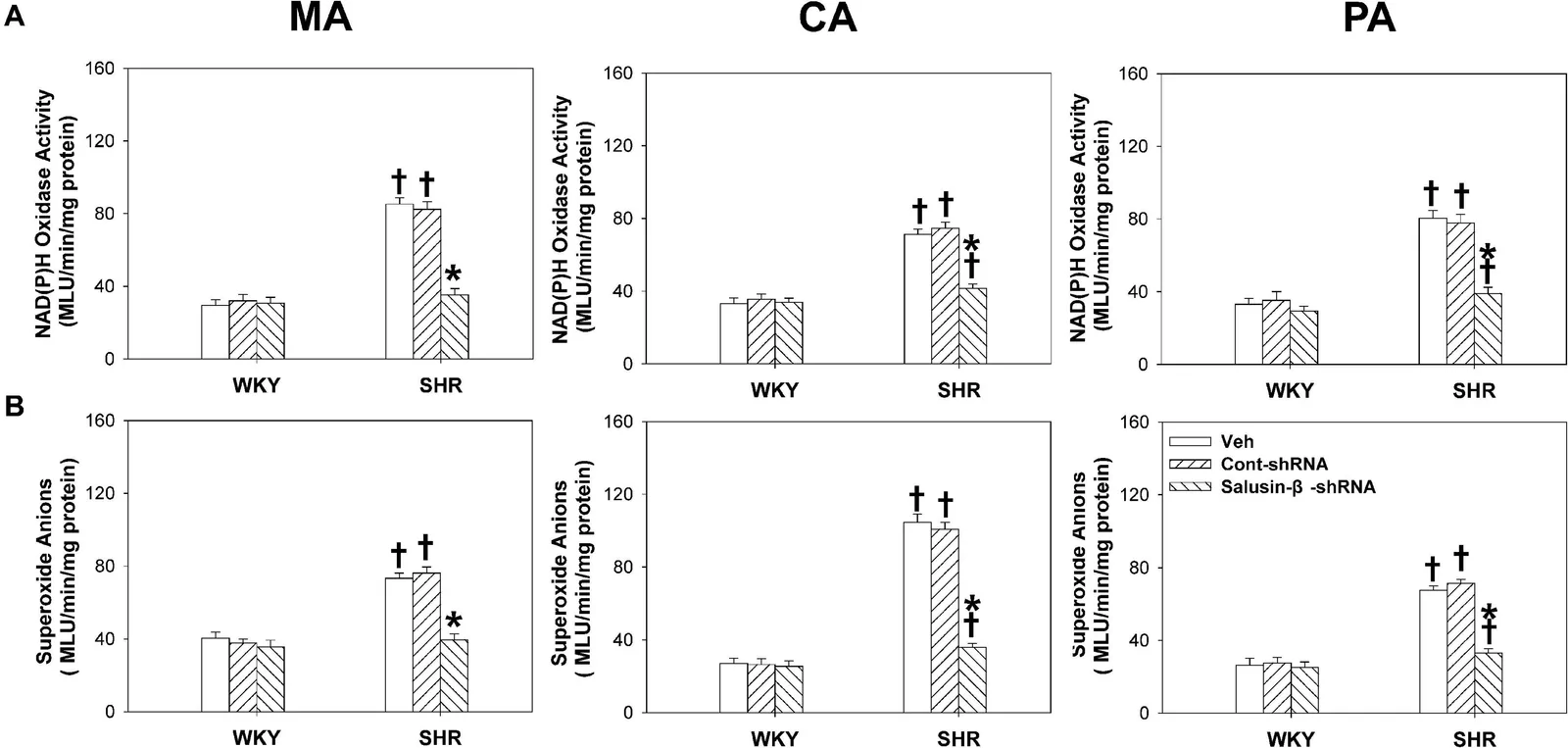
The effects of salusin-β knockdown on NAD(P)H oxidase activity **(A)** and superoxide anion levels **(B)** of MA, CA, and PA in WKY and SHR. Two-way ANOVA was used for data analysis, followed by Bonferrioni *post hoc* test to compare with the multiple groups. Values are mean ± SE. ^∗^*P* < 0.05 compared with Veh or Cont-shRNA, ^†^*P* < 0.05 compared with WKY. *n* = 6 for each group.

Compared with WKY rats, the NAD(P)H oxidase activity (Figure 8A) and superoxide anion levels (Figure 8B) of MA, CA, and PA in SHR were much higher and decreased significantly when salusin-β was silenced. However, knockdown of salusin-β had no significant effect on ROS production in WKY (Figure 8).

## Discussion

In hypertension, impaired vascular function, including increased vascular constriction and attenuated vascular relaxation of small arteries due to endothelial dysfunction, leads to continuous increase of peripheral resistance and the maintenance of hypertension. Impaired vascular function and subsequent vascular remodeling boost the progression of hypertension and organ damage (Brandes, 2014; Konukoglu and Uzun, 2017). The present study newly found that: (1) The plasma salusin-β level and protein expressions of salusin-β in MA, CA, and PA in SHR were much higher than that in WKY, which were decreased significantly by salusin-β knockdown; (2) The SBP, DBP, and HR of SHR were decreased by salusin-β knockdown, most significantly in DBP; (3) The high K^+^ solution-induced vasoconstriction in MA, CA, and PA was significantly enhanced in SHR and decreased by salusin-β knockdown; (4) Salusin-β knockdown significantly improved the impaired ACh-induced endothelium-dependent relaxations in MA, CA, and PA of SHR; this effect was almost blocked by NOS inhibitor L-NAME, but it was only influenced slightly by the K^+^ channels inhibitor TEA, the PGI_2_ synthesis inhibitor INDO, and NAD(P)H oxidase inhibitor VAS2870; (5) The eNOS level and activity as well as NO level of arteries in SHR were decreased, while NAD(P)H oxidase activity and superoxide anions level were increased in SHR, which were all redressed by salusin-β knockdown; and (6) Silencing salusin-β relieved vascular remodeling in SHR. These results suggest that the interference of salusin-β gene expression improves endothelium-dependent vasodilation, decreases vascular constriction and blood pressure, and repairs vascular remodeling of SHR by decreasing NAD(P)H oxidase generated ROS production and increasing eNOS activation to release NO.

It has been reported that salusin-β promotes the inflammatory response of human umbilical vein ECs (Karin and Delhase, 2000; Zhao et al., 2017), causes endothelial injury and dysfunction in diabetes mellitus (Sun et al., 2017; Zhu et al., 2017), and promotes the proliferation, migration and foam cell formation of VSMCs (Sun et al., 2015; Sun et al., 2016a,b). Intravenous administration of salusin-β in rats increases ABP, and overexpression of salusin-β in rats causes persistent elevation of blood pressure (Sun et al., 2015). As the exact natural receptor of salusin-β has not yet been found, gene silencing of salusin-β by shRNA is a good way to detect the effects of endogenous salusin-β (Sun et al., 2017). In the study, we found that after knockdown of salusin-β gene expression in rats, the SBP, DBP, and HR, especially DBP, of SHR decreased significantly. Compared to WKY, high K^+^ solution-induced arterial constrictions were enhanced significantly, and ACh-induced endothelium-dependent relaxation of arteries in SHR were significantly attenuated, which was in accordance with our previous findings (Zhang et al., 2019a). Salusin-β knockdown significantly decreased high K^+^ solution-induced vasoconstrictions and improved endothelium-dependent relaxations to a nearly normal physiological state in SHR. In addition, we found that compared with WKY, the lumen diameters of MA, CA, and PA in SHR decreased, while media thickness and media thickness/lumen diameter increased significantly, indicating the occurrence of vascular remodeling in SHR. After salusin-β knockdown, the lumen diameter, media thickness, and media thickness/lumen diameter of the MA, CA, and PA in SHR nearly returned to normal, suggesting that vascular remodeling in SHR was restored by salusin-β knockdown. These results showed that endogenous salusin-β plays a role in impairing vascular functions, maintaining high blood pressure, and inducing vascular remodeling in hypertension. We speculated that all these roles of salusin-β might attribute to its contribution to endothelial dysfunction in hypertension. Impaired vascular function, high peripheral resistance and blood pressure, and subsequent vascular remodeling are all consequences of endothelial dysfunction. It was also found that the salusin-β level in plasma and protein expression in the three arteries of SHR were significantly higher than those in WKY. This result indicated that the activity of salusin-β in the circulatory system in SHR increased, which might be an important cause of endothelial dysfunction in primary hypertension. Our results show that chronic gene interference with salusin-β expression greatly helps to improve endothelial function and decrease blood pressure, which suggests that salusin-β could be regarded as an important target in the prevention and treatment of hypertension. In addition, we also found that although salusin−β silencing decreased the ABP, SBP remained higher than the normotensive levels. We thought that there might be other factors besides salusin−β influencing the ABP in SHR, and silencing salusin−β partially decreased the ABP.

As we know, ACh stimulates ECs to release NO, PGI_2_, and EDHF. NO induces VSMCs relaxation by the GC-cGMP-PKG pathway (Schlossmann et al., 2003), EDHF causes smooth muscle relaxation by opening K^+^ channels (Miura et al., 1999), and PGI_2_ causes smooth muscle relaxation by cAMP pathway (Sprague et al., 2008). The question that remains is which factor mediates the improved effects of salusin-β knockdown on ACh-induced endothelium-dependent relaxations? In present study, we found that the NOS inhibitor L-NAME almost abolished the effect of salusin-β knockdown on ACh-induced arterial relaxation of SHR, while both the K^+^ channel inhibitor TEA and the PGI_2_ synthesis inhibitor INDO only played slight inhibitory roles in ACh-induced relaxations of SHR with salusin-β knockdown. This was also the case in SHR without salusin-β knockdown. These results indicated that the decrease in NO bioavailability played the most important role in the impaired endothelium-dependent relaxations in SHR and that the improved effects of salusin-β knockdown were achieved by increasing NO bioavailability. Our experiments found that the eNOS activity and level as well as NO production in MA, CA, and PA of SHR were significantly lower than those of WKY. After silencing salusin-β gene expression, the eNOS activity, eNOS level, and NO level in arteries of SHR were significantly restored, showing that these abnormalities in SHR were improved by salusin-β silencing. These results verified again that endogenous salusin-β contributes to endothelial dysfunction in hypertension by inhibiting the activation of eNOS and decreasing the release of NO. Some studies have indicated that NO derived from ECs might be an inhibitor of vascular remodeling in hypertension (Rudic and Sessa, 1999; Ghimire et al., 2017). This could also be the reason for the improved effect of salusin-β knockdown on vascular remodeling in SHR.

It has been reported that oxidative stress causes vascular endothelial damage, vascular contractive and diastolic dysfunction, and vascular remodeling in hypertension (Lee and Griendling, 2008; Dikalov and Dikalova, 2019). Studies have also reported that through the oxidative stress-related signaling pathway, salusin-β stimulates the migration of VSMCs and intimal hyperplasia after vascular injury (Aneja et al., 2008; Zhao et al., 2017). Salusin-β promotes foam cell formation and monocyte adhesion in atherosclerosis by stimulating the production of ROS (Sun et al., 2016a,b). Salusin-β provoked NAD(P)H oxidase derived-ROS production in human umbilical vein ECs (Panieri and Santoro, 2015; Sag et al., 2017; Yin et al., 2017; Esfahani et al., 2018). We intended to know whether ROS also mediated the effects of salusin-β in hypertension. In this study, we found that the NAD(P)H oxidase inhibitor VAS2870 improved endothelium-dependent arterial relaxations in SHR with Cont-shRNA, and it played similar roles as salusin-β knockdown in vascular relaxations in hypertension. This suggested that NAD(P)H oxidase derived-ROS are involved in endothelial dysfunction in hypertension. However, VAS2870 had no significant effect on endothelium-dependent vascular relaxation in SHR with salusin-β knockdown. We speculated that salusin-β knockdown already inhibited the NAD(P)H oxidase activity and VAS2870 could not serve any further action. The following results confirmed our conjecture. We further found that the NAD(P)H oxidase activity and superoxide anion levels in arteries of SHR were significantly higher than those of WKY and decreased significantly after knockdown of salusin-β. From these findings, we deduced that NAD(P)H oxidase-derived superoxide anions mediate the roles of salusin-β in regulating vascular function in SHR.

Studies have reported that ROS inactivate the eNOS and inhibit NO production (Forstermann and Li, 2011; He et al., 2019). We speculated that increased salusin-β in circulation of SHR first activates NAD(P)H oxidase on the EC membrane, and then induces an increase in ROS production. ROS, in turn, inhibits the activation of eNOS inside ECs and decreases the release of NO, resulting in a decrease in endothelium-dependent relaxation and an increase in vasoconstriction. These events contribute to the increase in peripheral resistance, maintenance of hypertension, and vascular remodeling. Salusin−β silencing in SHR revised this abnormality and then decreased ABP. However, both the salusin-β and ROS in normotensive WKY rats are at low levels, salusin−β silencing treatment might not play similar roles to increase eNOS activity and NO release as occurred in SHR. Therefore, salusin−β silencing had no significant effect on ACh-induced relaxations and blood pressure in WKY. In addition, there might be other possibilities for the inefficacy of salusin−β silencing in WKY. For example, salusin−β might have a different effect in WKY or the signaling pathway in arteries stimulated by salusin−β might be different between SHR and WKY.

## Conclusion

This study suggests that knockdown of salusin-β improved endothelium-dependent vascular relaxation and vascular remodeling and decreased the vasoconstriction and blood pressure in SHR, which might be accomplished by inhibiting NAD(P)H oxidase derived-ROS generation and increasing eNOS activation and NO release. Scavenging salusin-β improves vascular function and then prevents the development and progression of vasculopathy in hypertension, which has the potential to be a strategy for the treatment of hypertension in the future.

## Data Availability Statement

The raw data supporting the conclusions of this article will be made available by the authors, without undue reservation.

## Ethics Statement

The animal study was reviewed and approved by Nanjing Medical University Experimental Animal Care and complied with the Guide for the Care and Use of Laboratory Animals published by the US National Institutes of Health (NIH publication, 8th edition, 2011).

## Author Contributions

All authors contributed to the work in this manuscript. YH conceived and designed the experiments. YP, SS, and XW performed the experiments. SS, AC, WW, and XF analyzed the data. YH wrote the manuscript.

## Conflict of Interest

The authors declare that the research was conducted in the absence of any commercial or financial relationships that could be construed as a potential conflict of interest.

## Abbreviations

ACh: acetylcholine
ABP: arterial blood pressure
CA: coronary artery
EC: endothelial cell
eNOS: endothelial nitric oxide synthase
HR: heart rate
MA: mesenteric artery
MAP: mean arterial pressure
NO: nitric oxide
PA: pulmonary artery
ROS: reactive oxygen species
SBP: systolic blood pressure
SHR: spontaneously hypertensive rats
VSMC: vascular smooth muscle cell
WKY: Wistar-Kyoto rats.
