# Correction: Improvement of vascular function by knockdown of salusin-β in hypertensive rats via nitric oxide and reactive oxygen species signaling pathway

**DOI:** 10.3389/fphys.2026.2021867

**Published:** 2026-10-30

**Authors:** Yan Pan, Shuo Sun, Xingxing Wang, Aidong Chen, Xuejie Fei, Wei Wang, Ying Han

**Affiliations:** 1 Key Laboratory of Targeted Intervention of Cardiovascular Disease, Collaborative Innovation Center of Translational Medicine for Cardiovascular Disease, Nanjing Medical University, Nanjing, China; 2 Department of Physiology, Nanjing Medical University, Nanjing, China; 3 Department of Emergency, Shanghai Putuo District People’s Hospital, Tongji University, Shanghai, China

**Keywords:** hypertension, salusin-β, vascular function, nitric oxide, reactive oxygen species

There was a mistake in **Figure 1C** as published. The third representative image shown in **Figure 1C**, line 3, was inadvertently mistakenly pasted during figure preparation, it does not correspond to that experimental group described in the figure legend. This accidental figure error does not change the main findings, results, statistical analysis, or conclusions of the study. The corrected **Figure 1** appears below.

**Figure 1:**
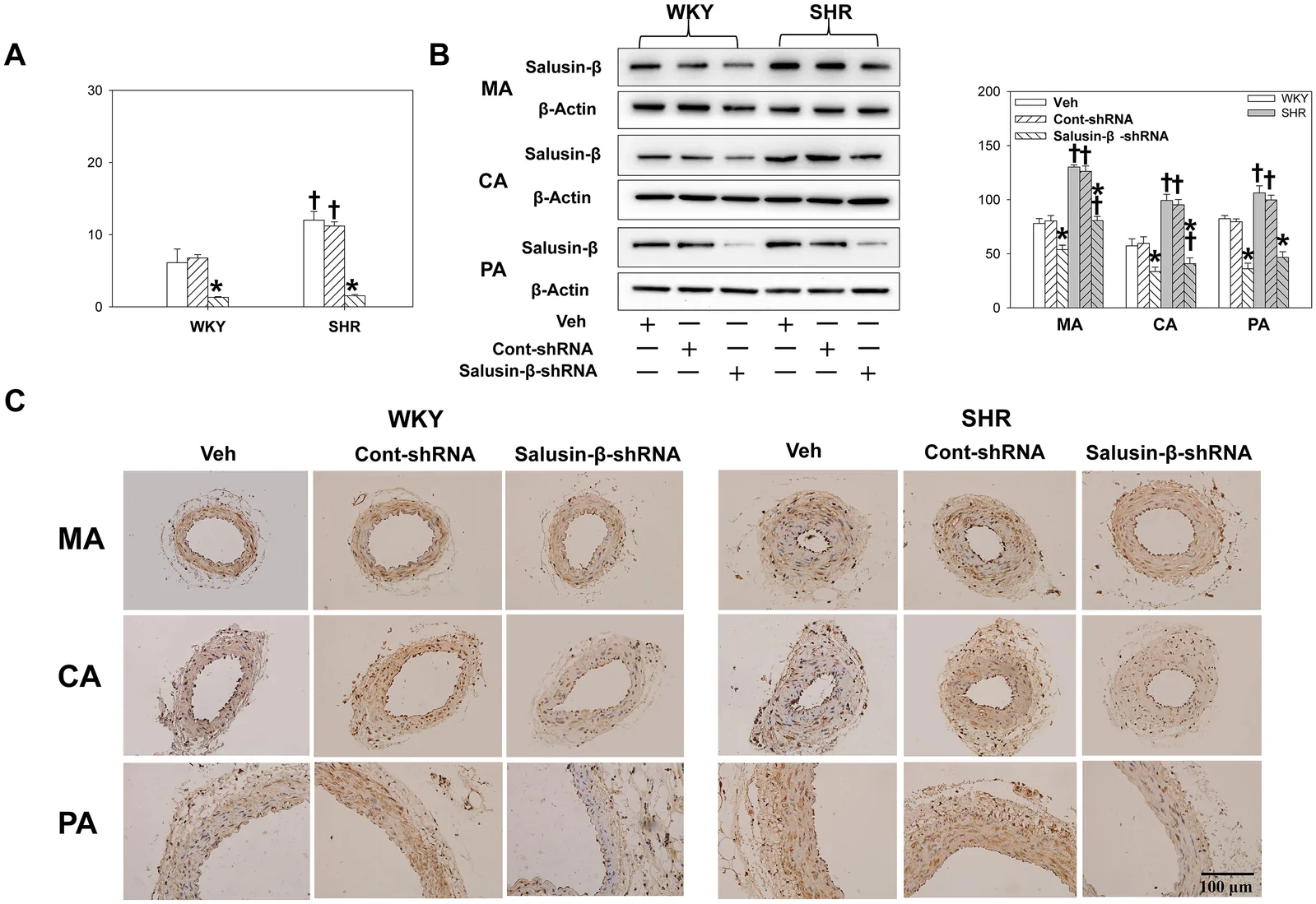
The salusin-β level in plasma **(A)**, and salusin-β protein expression of MA, CA, and PA in WKY and SHR detected by western blot **(B)** and detected by immunohistochemistry **(C)** after salusin-β knockdown. Two-way ANOVA was used for data analysis, followed by Bonferrioni post hoc test to compare with the multiple groups. Values are mean ± SE. *P < 0.05 compared with Veh or Cont-shRNA, ^†^P < 0.05 compared with WKY. n = 6 for each group.

The original version of this article has been updated.

